# The Expression of *LEP*, *LEPR*, IGF1 and *IL10* in Obesity and the Relationship with microRNAs

**DOI:** 10.1371/journal.pone.0093512

**Published:** 2014-04-01

**Authors:** Renata Viesti A. Collares, Wilson Salgado, Daniela Pretti da Cunha Tirapelli, José Sebastião dos Santos

**Affiliations:** 1 Department of Surgery and Anatomy of the Faculty of Medicine of Ribeirao Preto, University of São Paulo, Ribeirao Preto, SP, Brazil; 2 Department of Surgery and Anatomy of the Faculty of Medicine of Ribeirão Preto, University of São Paulo, Ribeirao Preto, SP, Brazil; 3 Department of Surgery and Anatomy of the Faculty of Medicine of Ribeirão Preto, University of São Paulo and Laboratory of Molecular Biology, Ribeirao Preto, SP, Brazil; Virginia Tech, United States of America

## Abstract

Obesity is a multifactorial disease, with epigenetic alterations. Have been described modifications in the expression of some microRNAs, and some proteins related to obesity. The objective was to determine and correlate, in obese patients, the gene expression of *LEP*, *LEPR*, *IGF1, IL10* and of miR-27a, miR-27b, miR-143 and miR-145. RNA was extracted from biopsies of subcutaneous fat, liver and visceral fat of 15 obese subjects submitted to bariatric surgery and of 15 non-obese subjects submitted to cholecystectomy for cDNA synthesis and for RT-PCR. The microRNAs were chosen using the TargetScan software. An increased expression of *LEP* and *IGF1* was detected in the subcutaneous fat of the obese group compared to control, while the expression of *IGF1* was higher in the control group than in the obese one. MiRNA-27a had a higher expression in the omentum of the obese patients and there was also a correlation in the expression of miRNA-145 and LEPR in the omentum of this group.

## Introduction

Obesity is a public health problem that has greatly increased over the last decade. It is accompanied by a series of diseases such as type 2 diabetes mellitus, cardiovascular diseases, and increased visceral fat, among others, which characterize the metabolic syndrome [1]–[3]. It is also characterized by an increased size and number of adipocytes and is considered to be a chronic inflammatory state since the concentration of many pro inflammatory cytokines increases with increased body fat [4], [5]. Adipose tissue is formed by different cell types and has an endocrine function since it secretes substances such as leptin (*LEP*), insulin-like growth factor 1 (*IGF1*) and interleukin 10 (*IL10*) [6]–[9].

Studies have shown that *LEP* is a protein hormone mainly synthesized by white adipose tissue which acts through a specific receptor and is directly related to the quantity of adipose tissue [10]. Obese individuals have hyperleptinemia and resistance to leptin, which is caused by changes in leptin receptors (*LEPR*s) as well as in its isoform or in deficiencies in the blood-brain transport system [11].

The study of the role of *IGF1* in cell metabolism has permitted us to associate it with obesity. This is a hormone that plays important roles in the organism, among them the regulation of adipose tissue mass, mediating the proliferation and differentiation of adipocytes during adipogenesis [12]. A study by Grégoire-Nyomba *et al.* [13] has suggested that both *LEP* and *IGF1* play a role in the regulation of body weight.

Another factor related to obesity is *IL10*, with a reduction of its concentration being related to the presence of metabolic syndrome [14]. *IL10* is an anti-inflammatory cytokine with an important role in the regulation of the immunological system by inactivating pro inflammatory cytokines through the suppression of macrophage function, with a consequent important role in the immunity of the gastrointestinal tract [15].

More recent discoveries have related changes involving some microRNAs to obesity. microRNAs are small non-protein coding RNAs consisting of 18 to 25 nucleotides which regulate gene expression in a negative and post-transcriptional manner [16], [17]. They have an important role in the regulation of various biological processes, including adipocyte differentiation, metabolic integration, insulin resistance, and appetite regulation, inhibiting the expression of target genes [18], [19].

Tang *et al*. [20] have suggested that miR-124a is involved in pancreatic cells, acting as a possible regulator of glucose in blood together with miR-375. In addition, it was demonstrated that both miR-143 and miR-145 are involved in cell differentiation [21], [22]. The microRNAs miR-143, miR-145, miR-27a and miR-27b were selected for inclusion in this study because, with the use of bioinformatics tool TargetScan (http://www.targetscan.org/), we verified that miR-27a and miR-27b had the genes of *LEP*, *IGF1* and *IL10* as targets. We also add the other microRNAs because some other studies showed that miR-143 interferes in glucose metabolism in mice, inducing insulin resistance, and that miR-145 is correlated with human body mass index [23], [24].

Study the possible relationship of miR-27a, miR-27b, miR-143 and miR-145 with leptin, leptin receptors, *IGF1* and *IL10* is important to try to understand the changes that occur in obesity and its physiopathology.

## Methods

The study was approved by the Research Ethics Committee of the University Hospital, Faculty of Medicine of Ribeirão Preto, University of São Paulo (HCFMRP/USP) and all patients gave written informed consent to participate (HCRP n° 8052/2010).

### Patients

The study was conducted on 15 obese patients with an indication of bariatric surgery according to the criteria of the International Federation for Surgery of Obesity, who were operated upon at HCFMRP/USP [25]. The control group consisted of 15 non-obese individuals submitted to video laparoscopic cholecystectomy at the State Hospital of Ribeirao Preto (HERP). Biopsies of subcutaneous tissue, liver and visceral fat were obtained from all subjects and snap frozen in liquid nitrogen (table 1).

**Table 1:** Characteristics of obese and control patients.

	OBESE	CONTROL
**Age (years)**	42.6	39.86
**Waist circunference (cm)**	131.4	78.13
**BMI (kg/m^2^)**	47.46	24.04
**Men**	2	3
**Women**	13	12

### RNA extraction

The RNA extraction was performed using Trizol reagent (Invitrogen, Carlsbad, CA, USA) according to the manufacturer's protocol. All RNA samples were quantified by spectrophotometry and examined by 1% agarose gel electrophoresis.

### Gene qPCR analysis

The system TaqMan Assay-on-demand (Applied Biosystems, Foster City, CA, USA) was used to analyze genes: LEPTIN (Assay ID Hs00174877_m1), LEPTIN RECEPTORS (Assay ID Hs00174497_m1), INTERLEUKIN 10 (Assay ID Hs00961622_m1) and INSULIN-LIKE GROWTH FACTOR 1 (Assay ID Hs00228005_m1). Complementary DNA (cDNA) was synthesized by reverse transcription using a commercial High Capacity cDNA Reverse Transcription Kit (Applied Biosystems, Foster City, CA, USA - #4368814) according to the manufacturer's manual (1 μg of RNA per sample), using a thermociclator for 30 minutes (16°C) to perform DNA denaturation, 30 minutes at 42°C for the strand extension, 5 minutes at 85°C to the enzyme deactivation and then, at 4°C. For the real time PCR, the conditions were pre-heating at 50°C for 2 μmin, denaturation at 95°C for 10 μmin, and 40 cycles of amplification and quantification (15 μs at 95°C and 1 μmin at 60°C). β-Actin was selected as endogenous control (housekeeping) for gene reactions, because it amplifies in all human tissues, and also it's used in other studies for its stability in the studied tissues [26], [27]. The mean values of Ct of β-Actin are showed on Table 2.

**Table 2:** Values of Ct B-ACTIN.

	Subcutaneous Fat	Visceral Fat	Liver
**Obese**	27.54	28.54	29.47
**Control**	31.58	26.86	28.67

### miRNA qPCR analysis

The cDNA was synthesized using 2.5 μng of RNA. The specific looped RT primers for miR-143, miR-145, miR-27a and miR-27b and reagents were included in the High Capacity c-DNA archive Kit (Applied Biosystems), and these components were incubated with 3.8 μU of RNase Inhibitor (Applied Biosystems) for 30 μmin at 16°C, 30 μmin at 42°C, 5 μmin at 85°C, and then held at 4°C. Real-time PCR analysis of cDNA was performed at 95°C for 10 μmin, followed by 40 cycles at 95°C for 15 μs and 60°C for 1 μmin in an ABI Prism 7500 Sequence Detection System using TaqMan Reaction Master Mix (Applied Biosystems), in accordance with the manufacturer's instructions. For such, we used the following primers: *has-miR-143, has-miR-145 has-miR-27a*, *has-miR-27b* (Applied Biosystems). The primers sequences are presented in table 3. The RNU24 and RNU48 genes were used as endogenous controls (housekeeping) for miRNA reactions.

**Table 3:** Primer sequences used for the microRNA's study.

cDNA primers	
GENE/miRNA	Primer sequences
**miR-27a**	UUCACAGUGGCUAAGUUCCGC
**miR-27b**	UUCACAGUGGCUAAGUUCUGC
**miR-143**	UGAGAUGAAGCACUGUAGCUC
**miR-145**	GUCCAGUUUUCCCAGGAAUCCCU

Data were analyzed (for both genes and miRNAs) using the ABI-7500 Sequence Detection System software and the variation of expression among samples was calculated by the 2^−ΔΔCt^ method.

### Statistical Analysis

Numerical variables between groups were compared with the Mann-Whitney test. Sperman's or Pearson's correlation test were used to verify the correlation between gene expression and microRNAs. Was used GraphPad PRISM software, version 5.0 (GraphPad Software Inc., San Diego, CA, USA) for statistical analysis. Significance was set at p<0.05.

## Results

The 15 obese patients had a BMI ≥ 36.8 μkg/m^2^ and an abdominal circumference ≥ 119 μcm and the 15 control subjects had a BMI ≤ 26.62 μkg/m^2^ and an abdominal circumference ≤ 87 μcm. The average expression and *p values* are shown in Tables 4 and 5, respectively.

**Table 4:** Mean numeric value (Fold) of the gene expression of some adipokines and microRNAs in the subcutaneous fat, omentum and liver of obese and control patients.

	OBESE			CONTROL		
	Subcutaneous Fat	Visceral Fat	Liver	Subcutaneous Fat	Visceral Fat	Liver
***LEP***	17.5489	15.9117	0.9657	1.8072	9.1492	0.8576
***LEPR***	0.7770	0.7306	0.7279	3.6288	8.4534	1.008
***IGF1***	11.0818	0.4146	0.7556	4.6619	1.3856	1.3070
***IL10***	2.5286	3.1246	2.0684	3.9530	1.6883	1.2991
**miR-27a**	3.4024	9.1012	2.5621	16.6941	1.5209	2.2145
**miR-27b**	8.3043	13.9761	3.6504	3.4932	2.3408	2.0524
**miR-143**	6.9401	7.3901	1.3379	9.6021	2.0974	1.9982
**miR-145**	3.7686	7.3600	1.4866	5.0547	1.7147	2.0671

**Table 5:** P values of the comparison of gene expression in the studied tissues between obese patients and the control group.

	Subcutaneous Fat	Visceral Fat	Liver
***LEP***	**<0.01**	0.21	0.43
***LEPR***	0.18	0.53	0.12
***IGF1***	**0.04**	**<0.01**	0.26
***IL10***	0.56	0.90	0.18
**miR-27a**	0.33	**<0.01**	0.47
**miR-27b**	0.43	0.27	0.40
**miR-143**	0.19	0.84	0.56
**miR-145**	0.10	0.98	0.74

### Gene expression

As shown in table 4, the gene expression of *LEP* in subcutaneous fat was significantly increased in the obese group compared to control (p = 0.00, Mann-Whitney test), whereas no difference was observed between groups in the expression of this gene in the liver and the omentum (p = 0.43; p = 0.21, respectively, Mann-Whitney test) (table 4). The gene expression of *LEPR* in subcutaneous fat, liver and visceral fat did not differ significantly between groups in the tissues tested (p = 0.18; p = 0.12; p = 0.53, respectively, Mann-Whitney test) (table 5).

The gene expression of *IGF1* was higher in the subcutaneous fat of the obese group compared to control (p = 0.04, Mann-Whitney test), and in the visceral fat of the control group compared to the obese group (p = 0.00, Mann-Whitney test). No significant difference between groups was observed in the liver (p = 0.26, Mann-Whitney test) (table 5).

The gene expression of *IL10* in subcutaneous fat did not differ between groups (p = 0.56, Mann-Whitney test). Similarly, its expression did not differ between groups in the liver and visceral fat (p = 0.18; p = 0.90, Mann-Whitney test) (table 5).

### microRNA expression

The expression of miR-27a was higher in the visceral fat of the obese group compared to control (p = 0.01, Mann-Whitney test) but did not differ between groups in subcutaneous fat (p = 0.33, Mann-Whitney test) or in the liver (p = 0.47, Mann-Whitney test) (table 5).

The expression of miR-27b in subcutaneous fat, liver and visceral fat did not differ between the obese and control groups (p = 0.43, p = 0.40, p = 0.27, respectively, Mann-Whitney test) (table 5).

The expression of miR-143 in subcutaneous fat, liver and visceral fat did not differ between the obese and control groups (p = 0.19, p = 0.56, p = 0.84, respectively, Mann-Whitney test) (table 5).

Similarly, the expression of miR-145 in subcutaneous fat, liver and visceral fat did not differ between the obese and control groups (p = 0.10, p = 0.74, p = 0.98, respectively, Mann-Whitney test) (table 5).

### Correlation

**Figure 1:**
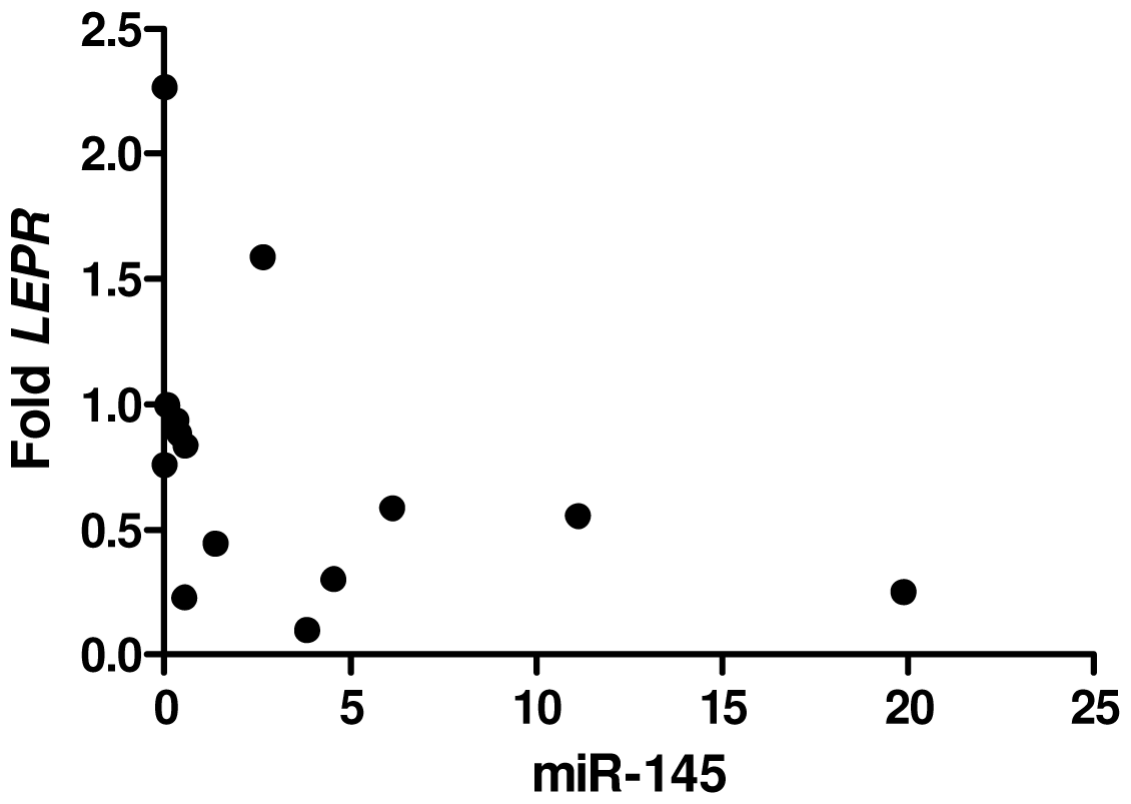
Correlation, in obese group, between the expression of miR-145 and *LEPR* in omentum. There was a negative correlation between microRNA expression and gene, confidence interval −0.8533 to −0.05087.

Was only observed a negative correlation between miR-145 and *LEPR* gene expression in the visceral fat of obese patients (r = −0.5780, and p = 0.0304) (figure 1). The p values for the correlations of the microRNAs with the genes, in the studied tissues, are showed on Tables 6, 7 and 8.

**Table 6:** P values of the correlation between micro RNAs and genes in the subcutaneous fat of the groups.

	miR-27a	miR-27b	miR-143	miR-145
***LEP***	0.72	0.24	0.26	0.35
***LEPR***	0.27	0.33	0.48	0.61
***IGF1***	0.73	0.07	0.09	0.06
***IL10***	0.36	0.43	0.83	0.83

**Table 7:** P values of the correlation between micro RNAs and genes in the liver of the groups.

	miR-27a	miR-27b	miR-143	miR-145
***LEP***	0.60	0.24	0.70	0.53
***LEPR***	0.12	0.33	0.10	0.31
***IGF1***	0.11	0.07	0.10	0.14
***IL10***	0.25	0.43	0.59	0.42

**Table 8:** P values of the correlation between micro RNAs and genes in the visceral fat of the groups.

	miR-27a	miR-27b	miR-143	miR-145
***LEP***	0.77	0.55	0.47	0.84
***LEPR***	0.52	0.09	0.97	0.03
***IGF1***	0.68	0.25	0.16	0.38
***IL10***	0.40	0.21	0.15	0.22

## Discussion

Obesity is a growing worldwide public health problem accompanied by health complications such as the development of type 2 diabetes mellitus, cardiovascular problems and sleep apnea, among others. Taken together, these factors characterize the metabolic syndrome [28]. The syndrome also involves genetic, hormonal, inflammatory, environmental and behavioral dysfunctions [28]. In order to contribute to the elucidation of the molecular pathways involved in the pathogenesis of obesity, we used real time PCR to quantify the expression of the genes *LEP*, *LEPR*, *IGF1* and *IL10* and of the microRNAs: miR-27a, miR-27b, miR-143 and miR-145 in the abdominal subcutaneous fat, the liver and omentum of individuals with morbid obesity of the central type compared to non-obese individuals.

The leptin (*LEP*) hormone is mainly synthesized in white adipose tissue and is related to the quantity of body fat. It acts on the control of food intake through specific receptors, (*LEPR*) located in the hypothalamus and in peripheral organs such as the liver. This hormone acts on the central nervous system, promoting reduced food intake and energy expenditure [29]. In a study on the blood of obese and lean individuals, Pardina *et al.* [30] observed higher *LEP* levels in morbidly obese individuals compared to lean individuals. The authors also reported that *LEP* levels were reduced one year after bariatric surgery, becoming identical to those of the control group. Esteghamati *et al*. [31] also observed that obese individuals with metabolic syndrome have higher leptin levels, suggesting that hyperleptinemia is associated with this syndrome.

In the present study, as expected, we observed a higher *LEP* expression in the subcutaneous fat of the obese group compared to control, whereas no difference was observed in the liver or omentum. The profiles of gene expression of subcutaneous abdominal fat and of the omentum are different, including the expression of some proteins, *LEP* among them [32]. Studies conducted on samples of abdominal subcutaneous fat and omentum have shown that the gene expression of *LEP* is higher in the abdominal subcutaneous fat, a result observed both in men and in women and in obese and normal weight individuals [33]–[37]. Since we did not detect a significant difference in *LEP* expression in the omentum of the obese group compared to control, we may assume that this was due to the fact that the highest production of *LEP* occurs in subcutaneous. In addition, Carmina *et al.* [38] observed that *LEP* expression decreases with increasing BMI both in the omentum and in the visceral subcutaneous fat. We may also suggest that another reason for the lack of a difference between groups in the expression of this gene in the omentum is that obese individuals have a lower mRNA production for this protein in the omentum and that this production is further reduced with increasing BMI.

Elevated circulating leptin concentration has been associated with obese patients with insulin resistance compared to normal weight individuals. In contrast, the circulating levels of leptin receptors are lower in obese individuals than in normal weight individuals. Also, the proportion of free circulating levels of *LEP* and *LEPR* is significantly higher in obese than in non-obese individuals [39]. Séron *et al*. [40] showed that expression of mRNA (long isoform) was elevated in subcutaneous fat of lean women and diminishes around 70% in the obese group. In visceral fat of both groups, the expression of this receptor is low. In the present study we did not detect a significant difference in the gene expression of *LEPR* in the obese group compared to control in any of the tissues analyzed. A possible explanation for this observation is the probably small number of individuals studied.

Obesity is also known to be associated with abnormal *IGF1* secretion. A 1997 study by Nam *et al*. [41] detected no significant difference in total *IGF1* between lean and obese individuals, but showed a higher free *IGF1* concentration in the obese group. Gomez *et al.* [42] observed a lower *IGF1* concentration in obese individuals, as also reported later by Pardina *et al*. [30]. The present results showed that *IGF1* is more expressed in the subcutaneous fat of obese subjects compared to control, whereas its expression is higher in the omentum of the control group. In the liver there was no difference in expression compared to control patients.

Another change detected in obesity is a chronic low grade inflammation, a state characterized by increased inflammatory markers. Literature data have demonstrated that circulating *IL10* levels are higher in obese women than in lean women and are lower in women presenting any component of the metabolic syndrome [14]. In another study, Bassols *et al.* [43] observed higher serum *IL10* concentrations in obese individuals compared to controls. Changes in circulating *IL10* concentration have been demonstrated in other studies in which polymorphisms of the promoter region of its gene reduce this concentration [44]. It has also been reported that rats with *LEP* and *IL10* deficiency have a lower body weight gain than rats having only the absence of *LEP* [45].

In the present study we did not detect a difference in *IL10* expression between groups. Since the obese group studied here had central obesity, which is considered to be a component of metabolic syndrome, we expected to find a lower expression in these individuals compared to control, as observed in the study by Esposito *et al*. [14]. The reduction of *IL10* associated with the presence of metabolic syndrome and type 2diabetes mellitus was also was observed by Van Exel *et al.* [46]. In view of the difficulty we had to find control subjects in the present study, since the samples were obtained from individuals with cholecystitis who have inflammation during a crisis, the fact that we did not obtain the expected result can be attributed to this situation.

Many studies have pointed out the participation of microRNAs in the regulation of metabolism and differentiation of adipose tissue. Klöting *et al*. [23], studying a cell culture of subcutaneous adipose tissue, observed that the expression of miR-27a was increased in individuals with type 2 diabetes mellitus, suggesting that this microRNA is related to adipose tissue dysfunction. Kim *et al.* [47] studied this same microRNA in rats and observed that its expression is increased in white adipose tissue of subcutaneous fat but is reduced during adipocyte differentiation. Studies have demonstrated that miR-27a is less expressed in the subcutaneous fat of individuals with type 2 diabetes mellitus [48]. In the present study we observed a higher expression of miR-27a in the omentum of the obese group compared to control.

Although there was a lower expression of miR-27a in the omentum of the obese group and no differences of the expression of *LEP* between groups, the correlation test didn't show any relationship between this microRNA and *LEP*, when there was expected a negative correlation, mainly because *LEP* is one of the targets of miR-27a.

Like miR-27a, miR-27b also participates in metabolism. Ji *et al.* [49] observed in a culture of rat hepatic stellate cells that these two microRNAs regulate in a negative manner the metabolism of fat and the proliferation of this cell type. In our study the expression of miR-27b did not differ between the group of obese individuals (subcutaneous fat, liver and omentum) and non-obese (control) individuals.

Still regarding microRNAs in obesity, increased miR-143 expression is known to be associated with increased body weight and with increased mesenteric fat [50]. A study carried out in order to determine the expression of this miR by microarray in samples of human subcutaneous adipose tissue demonstrated that its expression is increased in differentiating fat cells [48]. Another microRNA related to cell differentiation and proliferation is miR-145. La Rocca *et al.* [22] demonstrated that miR-145 expression is increased during cell differentiation. A 2009 study conducted on cell cultures of pre-adipocytes and differentiating adipocytes of obese individuals demonstrated that the expression of miR-145 was greatly reduced during cell differentiation [23].

In the present study, the expression of miR-143 and miR-145 did not differ between obese individuals (subcutaneous fat, liver and omentum) and non-obese controls. We expected to detect an increased expression of miR-143 and miR-145 in all tissues of the obese group compared to control since these microRNAs are related to increased cell differentiation and proliferation, but this was not the case. On the other hand, there was a negative correlation between the expression of miR-145 and *LEPR* in the omentum of obese patients.

In a critical evaluation of this article, the limited number of patients included in each group, mainly because of the limited grant dedicated to it, must have interfered in the statistical analysis. New study with more patients is now being planned and also with the possibility to include other microRNAs.

On the basis of the present data, we may conclude that there was a negative correlation between the expression of miR-145 and *LEPR* in the omentum of obese patients. This allows us to affirm that miR-145 may play a role in the regulation of obesity. New studies, with larger casuistic, are needed to confirm the real envolvement of the microRNAs with obesity.
